# Etiology of Myofascial Trigger Points

**DOI:** 10.1007/s11916-012-0289-4

**Published:** 2012-07-27

**Authors:** Carel Bron, Jan D. Dommerholt

**Affiliations:** 1 Scientific Institute for Quality of Healthcare, Radboud University Nijmegen Medical Centre, Nijmegen, The Netherlands; 2 Practice for Physical Therapy for Neck, Shoulder and Upper Extremity Disorders, Paulus Potterstraat 46, 9718 TK Groningen, The Netherlands; 3 Bethesda Physiocare Inc. and Myopain Seminars LLC, 7830 Old Georgetown Road, Suite C- 15, Bethesda, MD 20814-2440 USA; 4 Universidad CEU Cardenal Herrera, Valencia, Spain; 5 Shenandoah University, Winchester, VA USA

**Keywords:** Etiology, Myofascial, Pain, Trigger points, Muscle damage, Calcium, Sustained low-level contractions, Eccentric, Cinderella hypothesis

## Abstract

Myofascial pain syndrome (MPS) is described as the sensory, motor, and autonomic symptoms caused by myofascial trigger points (TrPs). Knowing the potential causes of TrPs is important to prevent their development and recurrence, but also to inactivate and eliminate existing TrPs. There is general agreement that muscle overuse or direct trauma to the muscle can lead to the development of TrPs. Muscle overload is hypothesized to be the result of sustained or repetitive low-level muscle contractions, eccentric muscle contractions, and maximal or submaximal concentric muscle contractions. TrPs may develop during occupational, recreational, or sports activities when muscle use exceeds muscle capacity and normal recovery is disturbed.

## Introduction

Myofascial pain syndrome (MPS) is described as the sensory, motor, and autonomic symptoms caused by myofascial trigger points (TrPs). TrPs are defined as exquisitely tender spots in discrete taut bands of hardened muscle that produce local and referred pain, among other symptoms. A TrP is composed of numerous so-called contraction knots. An individual contraction knot appears as a segment of a muscle fiber with extremely contracted sarcomeres and an increased diameter. The integrated TrP hypothesis postulates that in myofascial pain motor endplates release excessive acetylcholine, which is evidenced histopathologically by the presence of sarcomere shortening [1]. These areas of intense focal sarcomere contraction have been described in animals and humans.

An active TrP causes a clinical pain complaint. It is always tender and refers a patient-recognized pain on compression. It prevents full lengthening of the muscle, weakens the muscle, and mediates a local twitch response of muscle fibers when adequately stimulated. When compressed within the patient’s pain tolerance, it produces referred motor and often autonomic phenomena, generally in its pain reference zone [2]. A latent TrP is “clinically quiescent with respect to spontaneous pain; it is painful only when palpated. A latent TrP may have all the other clinical characteristics of an active TrP and always has a taut band that increases muscle tension and restricts range of motion” [2]. TrPs are very common, although literature about their prevalence is sparse [3–7].

Knowing the potential causes of TrPs is important to prevent their development and recurrence, but also to inactivate and eliminate existing TrPs. There is general agreement that any kind of muscle overuse or direct trauma to the muscle can lead to the development of TrPs. Muscle overload is thought to be the result of sustained or repetitive low-level muscle contractions, eccentric muscle contractions, and maximal or submaximal concentric muscle contractions [8]. Although muscle damage is not required for the development of TrP, there may be a disruption of the cell membrane, damage to the sarcoplasmic reticulum with a subsequent release of high amounts of calcium-ions, and disruption of cytoskeletal proteins, such as desmin, titin, and dystrophin. Ragged red (RR) fibers and increased numbers of cytochrome-c-oxidase (COX) negative fibers are common in patients with myalgia, which are suggestive of an impaired oxidative metabolism [9].

## Sustained Low-Level Contractions and Intramuscular Pressure

Mechanical muscle overuse can be defined as the result of muscle contractions that exceed muscle capacity. Since the capillary blood pressure ranges from approximately 35 mm Hg at the beginning (arterial side) to 15 mm Hg at the end of the capillary beds (venous side), the capillary blood flow is temporarily obstructed during muscle contractions. The blood flow recovers immediately with relaxation, which is consistent with its normal physiological mechanism. In dynamic rhythmic contractions, intramuscular blood flow is enhanced by this contraction-relaxation rhythm, also known as the muscular pump. During sustained muscle contractions, however, muscle metabolism is highly dependent upon oxygen and glucose, which are in short supply.

Even contractions performed at only 10 % and 25 % of capacity or maximum voluntary contraction (MVC) may produce intramuscular pressures high enough to significantly impair the intramuscular blood circulation. The association of the percentage of MVC and intramuscular pressure (IMP) is highly dependent upon the architecture of the muscle. It has been postulated that the percentage of MVC reported as the upper limit for localized muscle fatigue varies between muscles, because of the variation of IMP during a contraction. For example, 10 % of MVC of the supraspinatus muscle produces approximately 50 mm Hg of IMP, while 25 % of MVC of the trapezius muscle is good for only 22 mm Hg of pressure. Even lower values of IMP may affect muscle metabolism [10]. The EMG signs of localized muscle fatigue did not recover until the muscle contraction pressure was below 20 mm Hg in the biceps muscle [11]. According to Otten, the increased pressure gradients during low-level exertions may contribute to the development of pain [12] and eventually to the formation of TrPs (personal communication 2005).

Since oxygen and glucose are required for the synthesis of adenosine triphosphate (ATP), which provides the energy needed for muscle contractions, sustained contractions may cause a local energy crisis due to the lack of oxygen. To guarantee an adequate supply of ATP, the muscle can switch within a few seconds to anaerobic glycolysis. During the initial phase of glycolysis (sugar splitting) 1 glucose molecule is broken down into 2 pyruvic molecules releasing enough energy to form 2 ATP molecules. Under aerobic circumstances, oxygen reacts with pyruvic acid producing a high amount of ATP (16 molecules per pyruvic acid molecule), carbon dioxide and water. Under anaerobic circumstances, however, most of the pyruvic acid produced during glycolysis is converted into lactic acid, thereby increasing the intramuscular acidity (pH). Most of the lactic acid diffuses out of the muscle into the bloodstream; post-exercise lactic acid is washed out within 30 minutes after exercise. Unfortunately, when the capillary circulation is restricted, as in sustained low-level contractions, this process comes to a standstill.

Researchers at the US National Institutes of Health found that in the direct environment of active TrPs, the pH may be well below 5, which is more than sufficient to excite muscle nociceptors, including acid-sensing ion channels (eg, ASIC 1 and 3), and the transient receptor potential vanilloid receptor TRPV1 [13, 14]. Small increases of the H^+^ concentration, as seen with inflammation, heavy muscle work, and ischemia, are sufficient to excite muscle group IV endings, contributing to mechanical hyperalgesia and central sensitization [15]. Furthermore, a low pH downregulates acetylcholinesterase (AChE), increases the efficacy of acetylcholine (ACh), and maintains the sarcomere (super-) contraction. It also triggers the release of several nociceptive substances, such as calcitonin gene-related peptide (CGRP) [15], which can enhance the release of ACh from the motor endplate and simultaneously decrease the effectiveness of AChE in the synaptic cleft. CGRP also upregulates the ACh-receptors (AChR) at the muscle and thereby creates more docking stations for ACh. Miniature endplate activity depends on the state of the AChR and on the local concentration of ACh, which is the result of ACh-release, reuptake, and breakdown by AChE [12].

Relaxation within the muscle cells occurs when myosin-actin cross-bridges detach. After ATP is attached to the myosin molecule, the link between myosin and actin weakens, and the myosin head detaches from actin. In other words, the cross-bridge between myosin and actin ‘breaks’. Simultaneously, the Ca^2+^ ion detaches from the troponin molecule, which blocks tropomyosin. Under normal physiological circumstances, large amounts of free calcium-ions will reenter the sarcoplasmic reticulum by the Ca^2+^ pump (Calcium ATPase), which places a high demand on ATP during relaxation. In case of severe energy depletion, the sarcomeres may stay contracted, until enough ATP is available to resolve the intracellular Ca^2+^ accumulation. High concentrations of intracellular Ca^2+^ are associated with sustained sarcomere contraction and muscle damage. Ca^2+^ accumulation due to sustained motor unit activity has been suggested to play a causative role in the development of muscle disorders and TrPs [16].

In a preliminary study using Doppler ultrasound, Sikdar et al have shown that blood flow waveforms show significant differences between active TrPs, latent TrPs and normal sites. The flow waveforms near active sites showed increased systolic velocities and flow reversal with negative diastolic velocities [17•]. They identified 2 contributing factors, namely an increase in the volume of the vascular compartment, and an increased outflow resistance. Increased outflow resistance could be due to muscle contractures at the TrP that compress the capillary or venous bed. Sustained low-level contractions are common in the workplace where many occupations rely on prolonged postures, as seen in musicians, supermarket cashiers, computer operators, hairdressers, and dentists, among others.

## Dynamic Concentric Contractions

### Cinderella Hypothesis

Henneman, working with anesthetized cats, showed that in response to increasing physiological excitation, motor neurons are recruited in order of increasing size. This fundamentally important finding was verified in humans by Milner-Brown et al [18] and is now generally accepted. Smaller motor units have a smaller alpha-motor neuron cell body, smaller axons and fewer muscle fibers to activate compared with larger ones. According to the size principle, small motor units innervating type I red colored, slow oxidative fibers are recruited first, followed by red to pink colored, fast oxidative fibers, and finally by white colored, fast glycolytic fibers. Furthermore, small type I muscle fibers are activated during prolonged tasks [19], although a few studies have described some degree of motor unit substitution [20]. Hägg suggested that the continuous activity of these motor units in sustained contractions causes overuse muscle fiber damage, especially to the Type I fibers during low-level activities, which he summarized in his Cinderella hypothesis [21]. It is conceivable that in sustained low-level contractions and in dynamic repetitive contractions, ischemia, hypoxia and insufficient ATP synthesis in type I motor unit fibers are responsible for increasing acidity, Ca^2+^ accumulation, and subsequently sarcomere contracture. Furthermore, starting with the sarcomere (super-) contraction, the intramuscular perfusion slows down and ischemia and hypoxia will occur. This may lead to the release of several sensitizing substances causing peripheral sensitization [15, 22].

## Maximal or Submaximal Concentric Contractions

During (sub-) maximal concentric contractions high amounts of energy (ATP) are required. Initially, ATP is utilized from storage depots within the muscle fibers itself. After about 4–6 seconds, the muscle shifts to direct phosphorylation of ADP by creatine phosphate (CP). Phosphorylation produces ATP by coupling 1 phosphate group to an ADP molecule catalyzed by the enzyme creatine kinase. Stored ATP and CP provide enough energy for maximum muscle power for approximately 14–16 seconds. Hereafter, a short period of rest is needed to replenish the exhausted reserves of intracellular ATP and CP. When ongoing ATP demands are within the capacity of the aerobic pathway, muscular activity can continue for hours in well-conditioned individuals. However, when the demands of exercise begin to exceed the ability of the muscle cells to carry out the necessary reactions quickly enough, anaerobe glycolysis will contribute more and more of the total generated ATP. Finally, the muscle will run out of ATP and sustained sarcomere contractions may occur, starting the development of TrPs.

## Eccentric Contractions

During normal activity muscles are often active while being lengthened. This is referred to as an eccentric contraction [23]. Classic examples of this are walking, whereby the knee extensors are active while being lengthened just after heel strike while the knee flexes; placing an object gently downwards, whereby the arm flexors are active while being lengthened to control the rate of movement of the object. In other words, eccentric contractions are commonly used to control the rate of movement. In another scenario, the load on the muscle may increase to the point where the external force on the muscle is greater than the force that the muscle can generate. In that case, the muscle is forced to lengthen due to the high external load.

There is no solid evidence that eccentric loading would lead to the development of TrPs, but as Gerwin et al summarized, there is much overlap between the mechanisms of eccentric contraction and the development of TrPs [24]. In one study, Itoh et al found that healthy volunteers presented with tender ropy taut bands, which were painful on compression, immediately after 3 sets of eccentric exercises of the middle finger extensor muscle [25]. One day and 2 days after the exercises the findings were similar. After 7 days the muscles were recovered. While this study suggests that eccentric loading does contribute to the development of TrPs, the study also employed isometric loading in addition to eccentric loading, which precludes definitive conclusions.

There is evidence from biopsy studies that during eccentric contractions disruption of the cytoskeletal structures occurs, especially of the protein desmin that interconnects the adjacent myofibrils [26, 27] and of titin, the largest protein in the human body. Titin connects myosin filaments to the Z-bands and is also linked to actin filaments [28, 29]. Microscopic research revealed increased sections of abnormal fibers in eccentrically exercised muscles [30, 31], which appeared to be approximately 4 times the normal size [32]. This was only observed with eccentric exercise, and not with concentric exercise or passive stretching. Furthermore, all enlarged fibers were exclusively of the fast glycolytic type or fast contracting type II fibers that are highly fatigable. It is hypothesized that early in the exercise period fast glycolytic fibers fatigue as they are unable to regenerate ATP and as a result they enter into a rigor or high stiffness state. Subsequent stretching of the stiff fibers mechanically disrupts the fibers resulting in cytoskeletal and myofibrillar damage. There is also evidence that in eccentric exercised muscles the intracellular concentration of calcium is increased, probably because of disruption of the sarcoplasmic reticulum. As we have seen before high concentrations of Ca^2+^ keep myosin and actin molecules together. Furthermore, increased Ca^2+^ has the potential for activating several mechanisms that leads to further damage of the cell membrane and cytoskeletal disruption.

In contrast, Hocking has postulated that eccentric loading does not provide a good model for the TrP pathogenesis. He suggested that sustained partial depolarization or plateau depolarization of an α-motor neuron dendrites leads to lasting alterations in the function of the entire α-motor neuron [33] due to an upregulation of L- or N- type voltage dependent calcium channels and α1-adrenergic receptors and a downregulation of calcium-activated potassium channels, which would lead to an increase in the motor terminal cytosolic calcium ion concentration (personal communication, 2012). He suspects that the increased calcium concentration triggers the spontaneous release of ACh. In other words, according to Hocking, the increased ACh release would be the cause and not the result of the energy crisis. Alpha-1-adrenergic receptors are linked to L-type voltage dependent calcium channels, which would suggest that sympathetic activity would increase the cytosolic calcium ion concentration and the excessive release of Ach [34]. Rather than looking at overuse mechanisms, Hocking maintains that persistent nociceptive input causes the formation of TrPs through central sensitization of the C-fiber nociceptive withdrawal reflex and plateau depolarization of withdrawal agonist alpha-motor neurons and compensatory reticulospinal motor facilitation of antigravity muscles and plateau depolarization of withdrawal antagonist alpha-motor neurons [33]. Future research in the underlying mechanisms of TrP is much needed.

## Clinical Example

Eccentric contractions occur during all activities of daily life, but have been researched especially in sports. Overhead throwing, spiking in tennis and volleyball, javelin-throwing, downhill running, jumping and running (landing phase) involve eccentric activities. During the deceleration phase of throwing, the posterior shoulder muscles such as the infraspinatus, teres minor and major, posterior deltoid, and latissimus dorsi muscles, contract eccentrically not only to decelerate horizontal adduction and internal rotation of the arm, but also to resist shoulder distraction and anterior subluxation forces. A shoulder compressive force slightly greater than bodyweight is generated to resist shoulder distraction, while a posterior shear force of 40 %–50 % bodyweight is generated to resist shoulder anterior subluxation. Consequently, high activity is generated by the posterior shoulder musculature, in particular the rotator cuff muscles. The percentage of maximum voluntary isometric contraction (MVIC) is calculated in studies using needle electromyography. For example, the teres minor exhibits its maximum activity (84 % of MVIC), the infraspinatus 37 %, supraspinatus 51 %, posterior deltoid 69 %, latissimus 88 %, subscapularis 115 %, and triceps 89 % during the deceleration phase. Scapular muscles also exhibit high activity to control scapular elevation, protraction, and rotation during this phase. The lower trapezius muscle, which exerts force on the scapula in the direction of depression, retraction and upward rotation, generated its highest activity during the eccentric phase [35•]. High EMG activity from glenohumeral and scapular musculature during eccentric contraction illustrates the vulnerability of the posterior musculature for developing TrPs in the overhead-throwing athlete.

Posterior shoulder pain is a common complaint of throwing athletes and is mostly explained by posterior impingement [36, 37]. Interestingly, stretching exercises have been shown to assist in reducing the athlete’s pain and to normalize shoulder motion, particularly shoulder internal rotation and horizontal adduction. It is common for the overhead thrower to exhibit loss of internal rotation of 20 ° or more, referred to as “glenohumeral internal rotation deficit (GIRD).” This internal rotation deficit has been suggested to be a cause of specific shoulder injuries. Wilk expressed that loss of internal rotation is most often due to osseous adaptations of the humerus and posterior muscle tightness, and not posterior capsular tightness. Stretching exercises, including the sleeper’s stretch and supine horizontal adduction with internal rotation, are advised to improve the flexibility of the posterior musculature, which may become tight because of the muscle contraction during the deceleration phase of throwing [38]. TrPs can be found in many sportsmen, including elite volleyball players, swimmers, and runners [39–41].

## Conclusions

In spite of a lack of well-designed studies, the best available evidence supports that TrPs develop after muscle overuse. Several potential mechanisms may play a role, such as eccentric overload, submaximal sustained, and (sub)-maximal concentric contractions. A key factor is the local ischemia, which leads to a lowered pH and a subsequent release of several inflammatory mediators in muscle tissue. Hocking proposed an interesting counterargument, which deserves further exploration. Whether overuse mechanisms are the crucial initiating factor or persistent nociceptive input remains a point of debate and further study.
